# *Ex Vivo* Pharmacokinetics and Pharmacodynamics Modeling and Optimal Regimens Evaluation of Cefquinome Against Bovine Mastitis Caused by *Staphylococcus aureus*

**DOI:** 10.3389/fvets.2022.837882

**Published:** 2022-03-08

**Authors:** Li-jie Jiang, Xia Xiao, Ke-xu Yan, Tian Deng, Zhi-qiang Wang

**Affiliations:** ^1^College of Veterinary Medicine, Yangzhou University, Yangzhou, China; ^2^Jiangsu Co-innovation Center for Prevention and Control of Important Animal Infectious Diseases and Zoonoses, Yangzhou, China; ^3^Institute of Comparative Medicine, Yangzhou University, Yangzhou, China; ^4^Institutes of Agricultural Science and Technology Development, Yangzhou University, Yangzhou, China

**Keywords:** cefquinome, *S. aureus*, *ex vivo*, PK/PD modeling, cutoff, bovine mastitis

## Abstract

Cefquinome, the fourth-generation cephalosporin applied solely for veterinary medicine, is commonly used for bovine mastitis caused by *Staphylococcus aureus*. The present study aims to establish an optimal dose and provide a PK/PD Cutoff value (CO_PD_) for cefquinome against *S. aureus* based on *ex vivo* pharmacokinetics and pharmacodynamics (PK/PD) integration. This study investigated the pharmacokinetics (PK) of cefquinome when administered as three consecutive intramammary (IMM) doses of cefquinome in three healthy dairy cows at 75 mg/gland. Drug concentration was determined by HPLC-MS/MS assay. The *ex vivo* pharmacodynamics (PD) of cefquinome were evaluated by using a milk sample from a PK experiment. The relationship between the AUC/ MIC of cefquinome and bacterial loading reduction was simulated using a Sigmoid Emax model. The cefquinome concentration in milk attained a maximum level of 1.55 ± 0.21 mg/mL at 1.8 h after the third administration. The mean value of the area under the concentration-time curve (AUC_0−24_) was 26.12 ± 2.42 mg·h/mL after the third administration. The elimination half-life was 10.6 h. For PD profile, the MICs of cefquinome in milk were 2–4 times higher than those in the broth. *In vitro* time-killing curve shows that initial bacterial concentration has a huge impact on antibacterial effect on three strains. The antibacterial effect was weakened with the initial bacterial concentration increasing from 10^6^ to 10^8^ CFU/mL. The AUC_0−24h_/MIC index correlated well with *ex vivo* efficacy both for the initial inoculum of 10^6^ CFU/mL and 10^8^ CFU/mL (*R*^2^ > 0.84). According to the inhibitory sigmoid E_max_ model analysis, the PK/PD surrogate (AUC_0−24_/MIC) values were 8,638, 1,397, and 3,851 for bactericidal effect (*E* = −3) with an initial inoculum of 10^6^ CFU/mL, while the corresponding values were 12,266, 2,295, and 5,337, respectively, with the initial inoculum of 10^8^ CFU/mL. The *ex vivo* PK/PD based population dose prediction indicated a target attainment rate (TAR) of 90% of 55 mg/gland/12 h. The CO_PD_ for cefquinome against *S. aureus* was 2 μg/mL under the recommended dose of 55 mg/gland/12 h. However, it should be validated in clinical practice in future investigations. These results contribute to the rational use of cefquinome for mastitis treatment in clinical veterinary medicine.

## Introduction

Bovine mastitis is known as a serious disease in the dairy industry due to deterioration in the quality of milk, veterinary care expenses, and prohibitive labor costs for producers. Bovine mastitis usually results from bacterial, yeast, and even fungal or algae infection that accounts for almost 90% of all diagnoses (1). *Staphylococcus aureus* is one of the most common etiologic agents, which could result in chronic, contagious, and intractable bovine mastitis (2). *S. aureus* infection is extremely difficult to control (3), as it can release exotoxin, and survive in the intracellular where the drug concentration is often low. Until now, antimicrobial approaches have been the best way to control bovine mastitis. However, the resistance rate of *S. aureus* had been raised obviously and the number of multiple drug resistance (MDR) *S. aureus* has increased sharply, meaning the treatment of mastitis will become more difficult in the future (4, 5).

Cefquinome (CEQ) is a fourth-generation cephalosporin, solely for veterinary use. It has been used in bovine mastitis treatment for many years (6). It is highly stable when exposed to β-lactamases that are produced by the most clinically important bacteria. The pharmacokinetic (PK) characteristics of cefquinome have been studied in various animals, such as sheep, goats, cattle, buffalo calves, and camels via intravenous (i.v.) or intramuscular (i.m.) administration. The PK profiles of CEQ after local intramammary administration have also been performed in lactating cows and buffalo (7, 8). Previous reports have suggested that the intramammary recipe was more successful than systemic therapy, especially for mastitis caused by *S. aureus* (2, 9).

It has been reported that a rational antibacterial dose regimen based on pharmacokinetic and pharmacodynamic (PK/PD) modeling could balance the therapeutic effect and the emergence of resistance, and could also reduce the emergence and spread of drug resistance (10). PK/PD integration is also a key method to evaluate the clinically relevant relationship between time, drug concentration, and effect. The integration of the PK/PD model has now been widely applied in the evaluation of antibacterial activity and optimization of dosing regimens. In previous reports (11, 12) mouse or rat mastitis models were used for PK/PD evaluation in a mastitis *in vivo* PK/PD study, because establishing a mouse or rat mastitis model is easier than in other animals. However, this mastitis model cannot accurately reflect the mastitis in target animals such as a lactating cow. To date, few *in vivo* PK/PD model studies in cows have been reported. Although the PK fate of cefquinome and its efficacy in clinical treatment has been widely studied. There is no complete research linking the PK parameters to the PD effectiveness on mastitis therapy of various intramammary administration dosing regimens in dairy cows. It cannot be denied that there are differences between the *ex vivo* PK/PD model and the *in vivo* target animal intramammary infection model. The *ex vivo* model could not only ensure animal welfare but also save the cost of experimental intramammary infection in cows.

CO_PD_ is a significant parameter that assists in the definition of susceptibility breakpoints from the perspective of the exposure-response relationship (13). CO_PD_ was determined by Monte Carlo simulation (MCS), considering pharmacokinetic variation in target animals and PK/PD indices. This method has also been used by regulatory agencies such as CLSI and the European Committee on Antimicrobial Susceptibility Testing (EUCAST), in defining the susceptibility breakpoints (14).

In this study, the *ex vivo* PK/PD relationship of cefquinome against *S. aureus* in dairy cows through intramammary infusion was investigated. A 10,000-subject Monte Carlo simulation was performed to derive a PK/PD cutoff based on three aspects: MIC distributions of CEQ against *S. aureus*; pharmacokinetic/ pharmacodynamics (PK/PD) indices; and pharmacokinetics of CEQ in cows obtained. Then, a rational regimen of CEQ against dairy mastitis caused by *S. aureus* was determined.

## Materials and Methods

### Antimicrobial Agents

Cefquinome intramammary infusion was obtained from Merck Animal Health (Cobactan LC®; INTERVET). Cefquinome reference standard is purchased from Solarbio Life Sciences Co. Ltd. (Beijing, China). Test solutions of the antimicrobial agent were freshly prepared prior to use.

### Bacterial Strains and Animals

Sixty-three *S. aureus* strains isolated from clinical bovine mastitis individuals in Jiangsu China were evaluated in this study. ATCC 29213 was stored in our laboratory. B4030RH-31.4 and 2014RQG-33.15 were screened from 63 strains above. Brain-Heart-Infusion (BHI), Mueller-Hinton (MH) broth, and MH agar were purchased from Qingdao Hope Bio-Technology CO., Ltd. (Qingdao, China).

Three healthy Holstein dairy cows with weights ranging from 700 to 800 kg, were housed individually and fed with antimicrobial-free mixed ration and water *ad libitum*. Cows were milked twice daily during lactation.

### Antimicrobial Susceptibility

Susceptibility tests were determined according to the Clinical and Laboratory Standards Institute (CLSI) guidelines (15), using the following antibiotic: cefquinome (CEQ), streptomycin (STR), doxycycline (DOX), meropenem (MEM), ciprofloxacin (CIP), florfenicol (FFC), cefoxitin (FOX), tetracycline (TET), apramycin (APR), clindamycin (CLI), cloxacillin (CLO), and penicillin (PEN). CEQ MIC in milk was determined according to the CLSI micro broth dilution method, using milk as a bacteria medium. The MIC in milk was measured by sampling the mixture of drugs and bacteria and then 10-fold serial dilution and plating onto MH agar for colony counts calculation. The MIC in milk was defined as the minimum concentration where the bacteria loading equal to the initial concentration or grow <1 × log_10_ CFU/mL. The MIC_50_ and MIC_90_ values of CEQ were calculated, which represented the MIC value inhibiting the growth of at least the corresponding 50 and 90% of isolates. *S. aureus* American Type Culture Collection (ATCC) 29213 and *Escherichia coli* ATCC25922 strains were used as quality control. All determinations were performed in triplicate.

### *In vitro* Time-Killing Curves

An overnight culture in BHI broth of three *S. aureus* isolates including ATCC 29213, B4030RH-31.4, and 2014RQG-33.15 was diluted appropriately to attain two different concentrations of 10^6^ and 10^8^ CFU/mL. Pathogens were exposed to CEQ with different concentrations (0×, 0.5×, 1×, 2×, 4×, and 8× MIC) in MH broth and milk incubation at 37°C. An aliquot of 100 μL mixture was sampled at 0, 2, 6, 12, and 24 h and subjected to 10-fold serial dilution and then plated onto MH agar for visible counts calculation. The detection limit was 10 CFU/mL. All the MH agar plates were cultured at 37°C for 22 to 24 h before colony counting.

### Pharmacokinetics

The PK trials were performed in three healthy lactating bovines. CEQ was administered with 75 mg per udder every 12 h 3 times. A thirty second massage was applied to make the drug absorb homogeneously. After first intramammary (IMM) infusions, milk samples (about 50 mL at each time) were collected at time point: 0, 0.083, 0.167, 0.5, 1, 2, 4, 8, 12 h. After third IMM infusions, milk sample (about 50 mL at each time) were collected at the time points: 0, 0.083, 0.167, 0.5, 1, 2, 4, 8, 12, 24, 30, 36, and 48 h. The drug concentrations in milk were determined using the high-performance liquid chromatography-Mass Spectrometry (HPLC-MS/MS) method reported previously (16). Matrix matched calibration standards gave linear responses from 0.005 to 10 μg/mL (*R*^2^ > 0.999), with limits of quantification (LOQ) of 0.005 μg/mL. All samples with drug levels > 10 μg/mL were diluted proportionally with the extraction of blank milk. The PK parameters were calculated by the Winnonlin software (version 6.3, Pharsight, St. Louis, MO, USA).

### *Ex vivo* PK/PD Time-Killing Curve and PK/PD Analysis

*Ex vivo* time-killing curves were established using inactivated skimmed milk collected at specified time points from 0 to 84 h after IMM infusion administration. An overnight culture of *S. aureus* ATCC29213, B4030RH-31.4, and 2014RQG-33.15 were 10-fold diluted appropriately to attain two different concentrations of 10^6^ and 10^8^ CFU/mL. Pathogens were exposed to the milk sample of each time point after third administration and bred at 37°C. The method was the same as *in vitro* time-killing curves. The *ex vivo* antimicrobial effect (E) at a given CEQ concentration was expressed as the change in log_10_ CFU/mL after 24 h of incubation. The detection limit was 10 CFU/mL.

AUC_0−24h_/MIC values of CEQ against *S. aureus* were calculated for each sample. The effect (E) was the bacteria reduction during *ex vivo* time–killing assays. Data were analyzed using the sigmoid E_max_ model (17, 18). WINNONLIN software as follows:

$$\begin{array}{l} E=E_{0}-\frac{E_{MAX}\times C_{e}^{N}}{EC_{50}^{N}+C_{e}^{N}} \end{array}$$

where E_0_ is the change in log10 CFU/mL after 24 h incubation in the control sample (absence of CEQ) compared with the initial inoculum; *E*_max_ is the difference in effect between the greatest amount of growth (as seen for the growth control, E_0_) and the greatest amount of kill; *C*_e_ is the AUC_0−24_/MIC in the effect compartment; EC_50_ is the AUC_0−24_/MIC value producing a 50% reduction in bacterial counts from the initial inoculum, and N is the Hill coefficient that describes the steepness of the AUC_0−24_/MIC-effect curve.

### Monte Carlo Analysis and Determination of Pharmacodynamics Cutoff Value

A Monte Carlo simulation (MCS) with 10,000 iterations was conducted using Crystal Ball software (version 7.2.2) (Oracle, United States) based on MIC distribution, PK parameters for CEQ in milk after IMM infusion, indices of PK/PD targets obtained in the *ex vivo* PK/PD modeling (19–22). The MIC data of CEQ against *S. aureus* were collected according to the previous study reported data (23–27), and its distribution was shown in Supplementary Figure S1. The AUC_0−24_ was assumed to be log-normally distributed for the mean values and confidence intervals (CI). The distribution of the AUC_0−24*h*_/MIC was calculated through MCS. Then a target value of Bactericidal activity AUC_0−24_/MIC derived from *ex vivo* PK/PD modeling was set to calculate the target attainment rate (TAR) of the corresponding dosing regimen. CO_PD_ was defined as the MIC at which the PTA reached up to 90% under an existing dose or a recommended dose regimen, according to the CLSI guidelines described in previous reports by Turnidge and Paterson (28) and Zhang et al. (29).

## Results

### MIC Results

**Figure 1:**
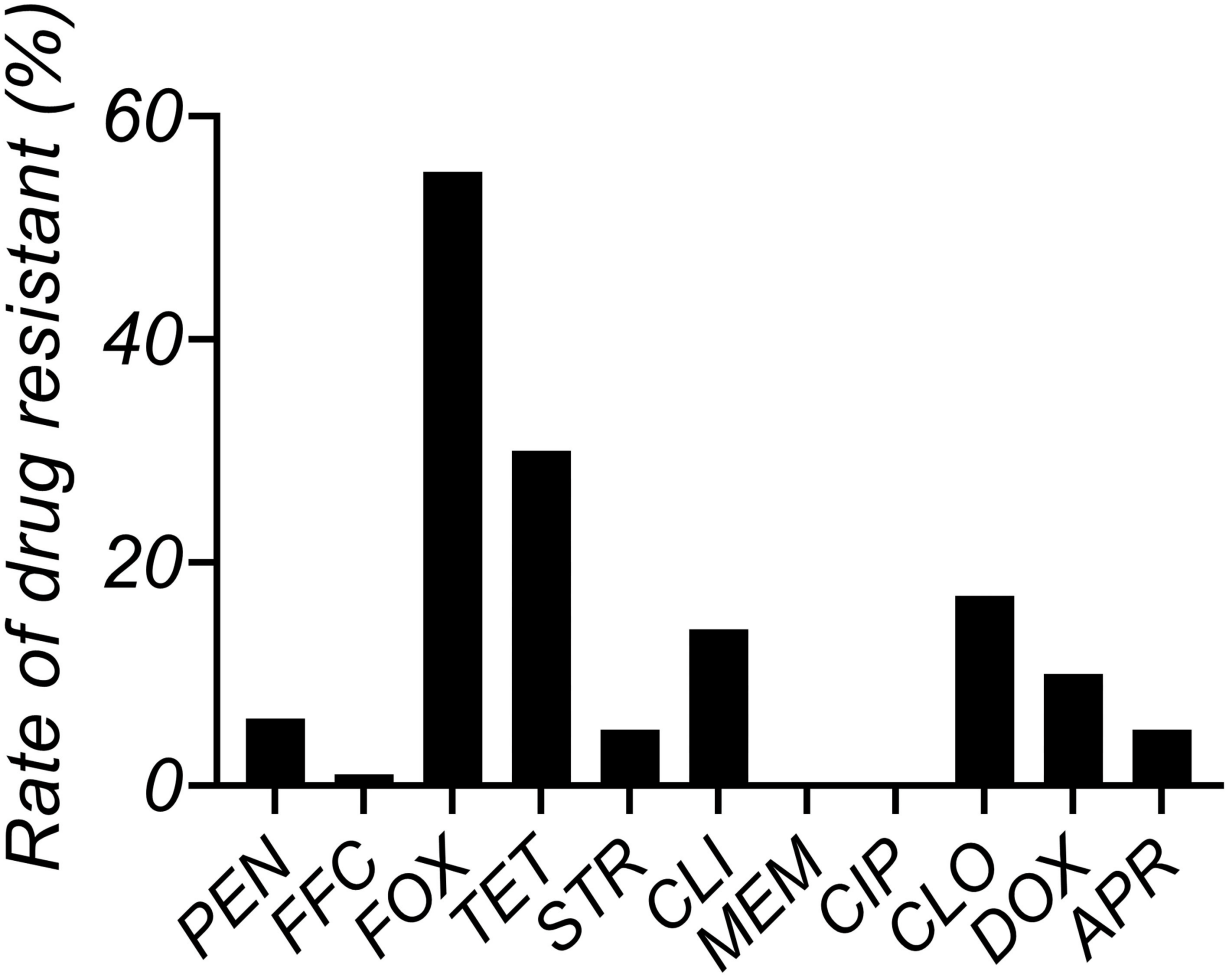
*In vitro* susceptibility assays of 63 *Staphylococcus aureus* isolates used in this study.

The MIC distributions of antimicrobials against *S. aureus* (n = 63) isolated from clinical mastitis in Jiangsu China are shown in Supplementary Table S2. The MIC_50_ and MIC_90_ values of CEQ are 0.5 and 2 μg/mL in MH broth and 1 and 2 μg/mL in milk. MIC_90_ of CEQ in milk was one dilution higher than their MIC_50_. The resistance rate of tetracycline was most serious, attaining 30%, and the resistance rate of FOX and CLO was as high as 17% (Figure 1).

The MICs of CEQ in milk were two to four times more than in MH broth. The MIC of CEQ in the broth for B4030RH-31.4, 2014RQG-33.15, and ATCC 29213 were 4, 1, and 0.5 μg/mL, respectively, and in the milk they were 16, 2, and 1 μg/mL, respectively (Supplementary Table S3).

### *In vitro* Time-Killing Curves

**Figure 2:**
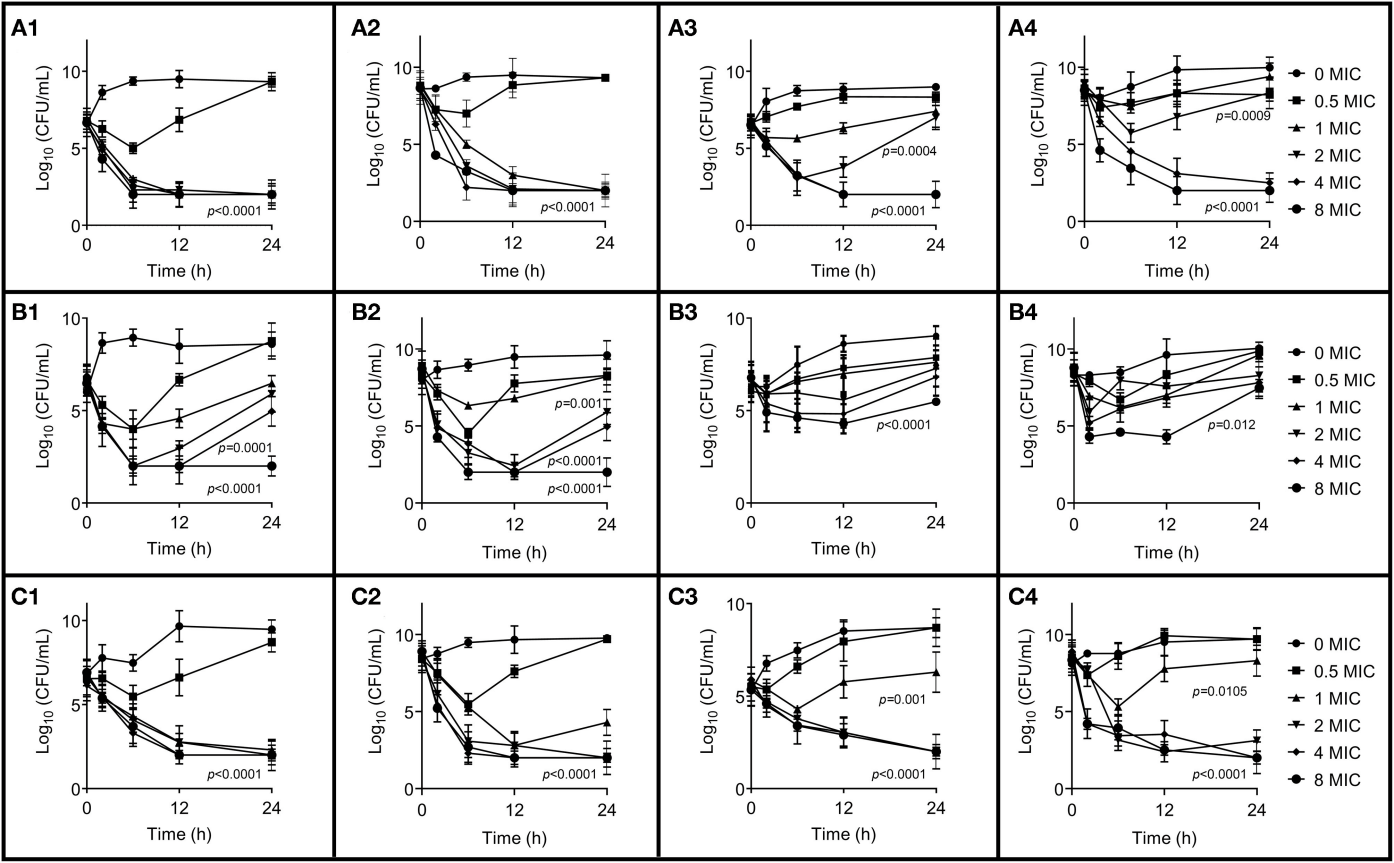
*In vitro* time-killing curves of cefquinome in broth and milk against *S. aureus* ATCC 29213, B4048RH-31.4, and 2014RQG-33.15, respectively. **(A1**–**A4)** Represents the *S. aureus* ATCC 29213, **(B1–B4)** represents the *S. aureus* B4048RH-31.4, and **(C1–C4)** represents the *S. aureus* 2014RQG-33.15. **(A1,B1,C1)** Show antibacterial activity against an initial bacterial load of 10^6^ log_10_CFU/mL in broth, and **(A2,B2,C2)** show antibacterial activity against t initial bacterial load 10^8^ log_10_CFU/mL in broth. **(A3,B3,C3)** Show antibacterial activity against an initial bacterial load of 10^6^ log_10_CFU/mL in milk, and **(A4,B4,C4)** show antibacterial activity against an initial bacterial load of 10^8^ log_10_CFU/mL in milk. Error bars represent the SD (*n* = 3) and *p*-value were calculated by Student's *t*-test.

The *in vitro* time-killing curves against *S. aureus* ATCC 29213, B4048RH-3.14, and 2014RQG-33.15 with two initial bacterial loads in artificial medium and milk are shown in Figure 2. Bactericidal activity was observed in the broth at 1 × MIC CEQ concentration against ATCC 29213 with bacteria titer of 10^6^ and 10^8^ log CFU/mL (Figures 2A1,A2), but the concentration of CEQ was 4 × MIC in the milk matrix, which could achieve the same effect (Figures 2A3,A4). The bactericidal activity was observed at 8 × MIC against B4030RH-3.14 in the broth (Figures 2B1,B2). However, in milk, CEQ cannot inhibit the growth of the same strain B4030RH-3.14 even at 8 × MIC (Figures 2B3,B4). The drug killing mode against 2014RQG-33.14 was similar to ATCC29213. The bactericidal was achieved at 1 × MIC CEQ concentration in broth (Figures 2C1,C2), while the corresponding concentration was 2 × MIC in milk against 2014RQG-33.14 (Figures 2C3,C4).

### Milk Pharmacokinetics for CEQ

**Figure 3:**
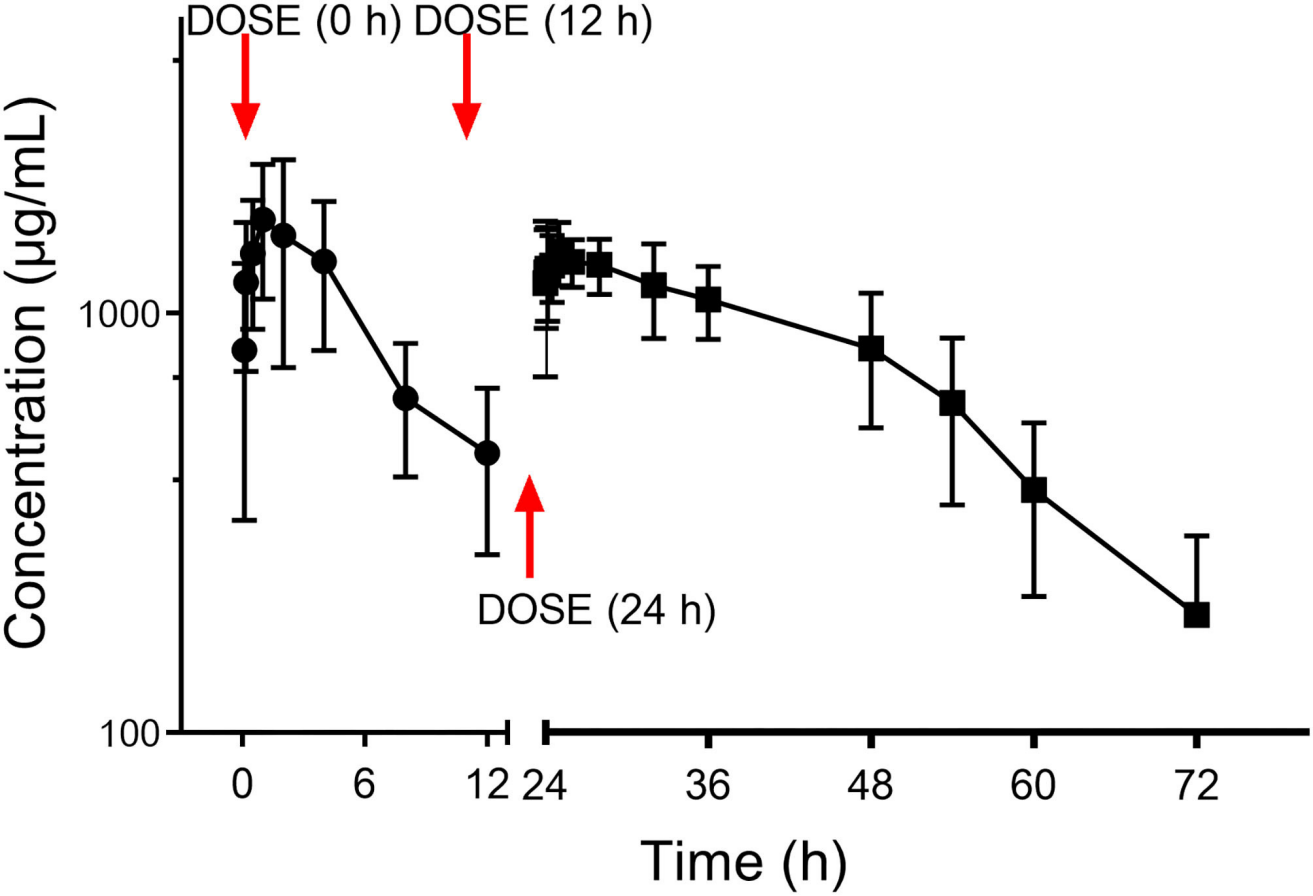
Milk concentration vs. time curve of cefquinome following three consecutive IMM administration of 75 mg/gland.

The PK curve of CEQ in milk is shown in Figure 3. After the first CEQ IMM administration, the CEQ concentration in milk attained a maximum level of 1.80 ± 0.64 mg/mL at 1.71 h. The AUC_0−12_ was 15.53 ± 5.64 mg·h/mL. Then, after the third IMM administration, the CEQ concentration in milk attained a maximum level of 1.55 ± 0.21 mg/mL at 1.8 h. The drug elimination half-life was 10.6 ± 4.01 h, the AUC_0−24_ was 26.12 ± 2.42 mg·h/mL. The mean residue time was 22.58 ± 7.83 h (Table 1). These results suggested that CEQ was eliminated slowly and maintained a long period of effective concentration in milk.

**Table 1:** Pharmacokinetic parameters of cefquinome in milk following the administration of three consecutive IMM of 75 mg cefquinome in each breast of 3 cows (Mean ± SD, *n* = 12).

**Parameters**	**Unit**	**Dosage (75 mg/gland)**	
		**First**	**Third**
C_max_	mg/mL	1.80 ± 0.64	1.55 ± 0.21
T_max_	h	1.71 ± 1.18	1.8 ± 2.93
AUC_last_	mg·h/mL	15.53 ± 5.64	42.25 ± 12.3
AUC_0−24_	mg·h/mL	–	26.12 ± 2.42
MRT	h	–	22.58 ± 7.83
CL/F	L/kg	–	2.07 ± 0.38
Vz/F	L/h/kg	–	31.09 ± 11.04
T_1/2β_	h	–	10.6 ± 4.01

### *Ex vivo* PK/PD Integration

**Figure 4:**
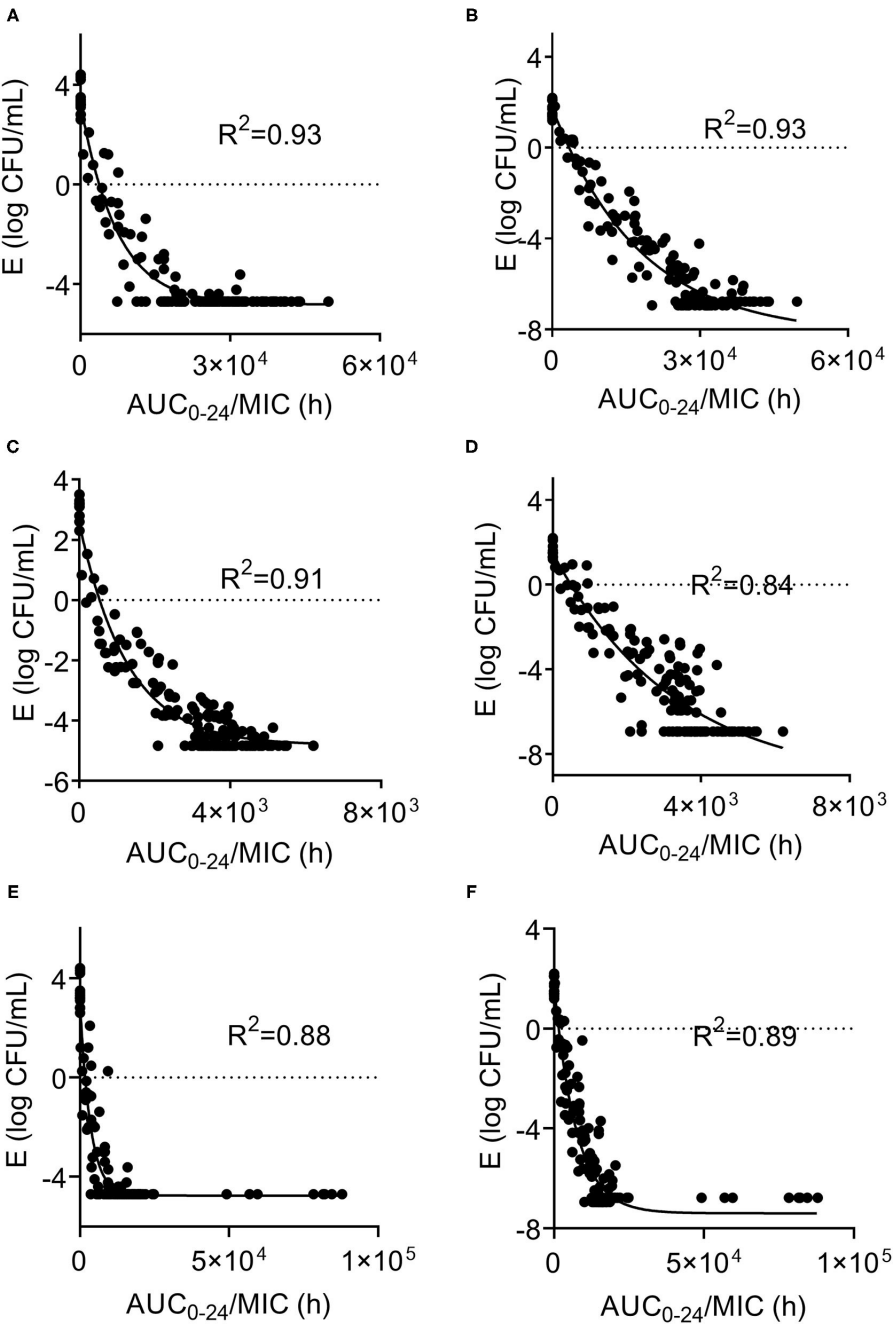
Sigmoid E_max_ relationships between *ex vivo* antibacterial effect and PK/PD indexes of cefquinome against *S. aureus* ATCC29213, B4048RH-31.4, 2014RQG-33.15. **(A,B)** Showed Sigmoid E_max_ relationships against *S. aureus* ATCC29213 with an initial bacterial load of10^6^ and 10^8^ log10CFU/mL. **(C,D)** showed Sigmoid E_max_ relationships against *S. aureus* B4048RH-31.4 with an initial bacterial load of 10^6^ log_10_CFU/mL and 10^8^ log_10_ CFU/mL. **(E,F)** Showed Sigmoid E_max_ relationships against *S. aureus* 2014RQG-33.15 with an initial bacterial load of 10^6^ log_10_CFU/mL and 10^8^ log_10_CFU/mL.

In the *ex vivo* time-killing curve, bacteria number changes with the drug concentration of the milk sample. The higher the drug concentration, the stronger the bactericidal activity. The initial inoculum concentration will also affect the antibacterial activity of CEQ, that is, the bactericidal effect will be reduced under high initial inoculum amount (10^8^ log CFU/mL). The CEQ antimicrobial activity is different when in a different medium. The relationships between PK/PD parameters and the *ex vivo* antibacterial effects in milk with different initial titers of *S. aureus* (1 × 10^6^ CFU/mL, 1 × 10^8^ log CFU/mL) are shown in Figure 4. According to Figure 4, the bactericidal effect of CEQ in milk is weak compared to in the broth. The AUC_0−24h_/MIC index correlated well with *ex vivo* efficacy with the initial bacteria loading of 10^6^ cfu/mL (*R*^2^ > 0.88). While for the initial bacteria loading of 10^8^ cfu/mL, the *R*^2^ was about 0.84, indicating that bacterial loading, in other words, the severity of infection poses an effect on the PK/PD modeling results. The PD parameters of E_0_, E_max_, and PK/PD parameters required for various degrees of antibacterial activity and the Hill coefficient N are presented in Table 2. According to the inhibitory sigmoid E_max_ model analysis, the AUC_0−24_/MIC were 8,638, 1,397, and 3,851 to attain bactericidal effect against ATCC 29213, B4030RH-31.4, and 2014RQG-33.15 with the initial inoculum of 10^6^ CFU/mL, while the corresponding AUC_0−24_/MIC values were as high as 12,266, 2,295, and 5,337 with the initial inoculum of 10^8^ CFU/mL.

**Table 2:** Integration of *ex vivo* PK/PD after administration of three consecutive IMM of cefquinome against in *S.aureus*.

**Parameter**	**ATCC 29213**		**B4048RH-31.4**		**2014RQG-33.15ATCC29213**	
	**10^**6**^CFU/mL**	**10^**8**^CFU/mL**	**10^**6**^CFU/mL**	**10^**8**^CFU/mL**	**10^**6**^CFU/mL**	**10^**8**^CFU/mL**
Emax	9.61	11.35	8.4	9.144	9.01	9.13
EC_50_	5,437	15,489	1,040	1,158	2,263	5,405
E_0_	3.2	1.6	3.6	2.2	3.2	1.41
Bacteriostatic activity	3,172	5,177	974	1,564	1,695	2,138
Bactericidal activity	8,638	12,266	1,397	2,295	3,851	5,337
Bacterial elimination	12,691	15,227	1,734	2,608	5,665	6,886
*N*	1.29	1.65	4.4	3.3	1.75	1.15

### Dose Assessment and PK/PD Cutoff Determination

The MIC values of CEQ against *S. aureus* were collected from previously reported data (23–27) and this study. The MIC distribution of CEQ against *S. aureus* is shown in Supplementary Figure S1, MIC_50_ and MIC_90_ were 1 and 2 μg/mL, respectively. Through Monte Carlo simulation, the optimal dosage for slight infection mastitis (10^6^ CFU/mL) to bactericidal (*E* = −3) activity against *S. aureus* was 38 mg/gland/12 h, and for severe infection mastitis (10^8^ CFU/mL) for bactericidal (*E* = −3) activity against *S. aureus* it was 55 mg/gland/12 h (Table 3).

**Table 3:** Predicted dose of cefquinome against *S.aureus* to reach 50%TAR, 90%TAR under different bacterial load (mg/gland/12 h).

**Predicted dose (mg)**		**Target rations (10** ^**6**^ **)**		**Target rations (10** ^**8**^ **)**	
		**50%**	**90%**	**50%**	**90%**
29213	Bacteriostatic (*E* = 0)	7	14	11	23
	Bactericidal (*E* = −3)	18	38	26	55
	Eradication (*E* = −4)	27	57	31	70
B4048RH-31.4	Bacteriostatic (*E* = 0)	34	36	54	57
	Bactericidal (*E* = −3)	49	51	79	87
	Eradication (*E* = −4)	60	63	91	98
2014RQG-33.15	Bacteriostatic (*E* = 0)	3	8	4	10
	Bactericidal (*E* = −3)	8	17	11	24
	Eradication (*E* = −4)	12	25	15	31

**Figure 5:**
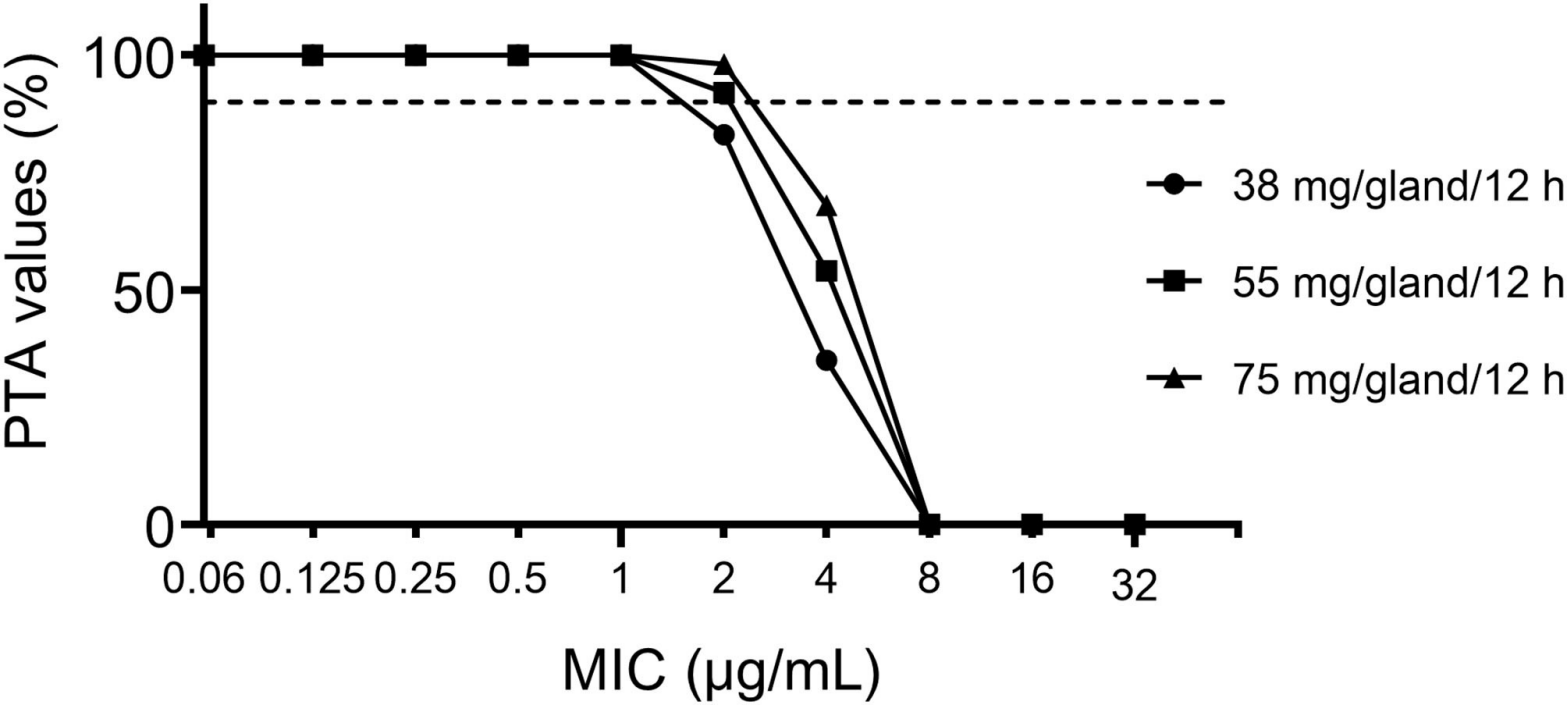
Probability of target attainment (PTA) of cefquinome against bovine mastitis caused by *S. aureus* at the dose of 38, 55, 75 mg/gland/12 h.

The PTAs following CEQ administration at a dose of 38, 55, 75 mg/gland/12 h are shown in Figure 5. For the recommended dose and clinical recommended dose (38, 55, 75 mg/gland/12 h), the PTA > 90% could only be achieved for MIC <2 or 1 μg/Ml, which means, the PK/PD cutoff for CEQ against *S. aureus* was 1, 2, 2 μg/mL, respectively. The recommended dose of 55 mg/gland/12 h was lower than 75 mg/gland/12 h, which not only reaches bactericidal effect even in severe infection mastitis but reduces the risk of drug resistance. Therefore, the recommended dose regimen of 55 mg/gland/12 h in this study is the optimal dose regimen for clinical mastitis therapy.

## Discussion

According to previous research, the PK/PD modeling of CEQ against mastitis was studied in the mouse mastitis model (30) instead of bovine. To our knowledge, this study is the first to address the PK/PD modeling and CO_PD_ of CEQ against *S. aureus* in lactating cows. In this study, we investigated pharmacokinetic and *ex vivo* PK/PD modeling of CEQ after IMM administration in healthy cows, and the rational dose regimen and CO_PD_ were evaluated.

According to the MIC result, PEN, CLO, and CLI are common use drugs in the clinical treatment of most dairy farms for mastitis. This could be the reason why the resistance rate is higher than other drugs. However, TET displays the highest resistance rate except for FOX. The possible reason for this is high-frequency TET-resistant gene horizon transfer and low fitness cost in *S. aureus* (31–33). The result of MIC in milk is different from in broth, and most of the MIC data in milk were two to four times higher than those in broth. Milk as the culture medium could provide a bacterial growth environment, especially for mastitis-related bacteria. Data of MIC in milk can better reflect the real situation in the bovine gland. As indicated in Figure 2, CEQ displayed a discrepancy in antimicrobial effect because of different mediums for the same strain. The high bacterial load group (8 log_10_CFU/mL) exhibited a weak bactericidal effect in the same medium. Although a 6 log_10_CFU/mL bacteria loading is usually used as the initial inoculum *in vitro* killing trials, in this study bacterial loads of 6 and 8 log_10_CFU/mL were employed to simulate slight and severe mastitis. In this study, significantly, CEQ bactericidal effect against *S. aureus* was influenced along with the bacterial initial load. In the high bacterial concentration (8 log_10_CFU/mL) group, CEQ bactericidal effect is weaker compared to normal bacterial concentration (6 log_10_CFU/mL) group. The amount of initial load will contribute to a determining factor. The reason is that when the initial concentration of *S. aureus* was high, the reproduction was low due to the limit of space, nutrition, and so on. The result suggested that the milk as a medium could show the real drug effect better than in broth. Compared with previous work (30), wild *S. aureus* strain including B4030RH-31.4 and 2014RQG-33.15 in the *in vitro* time-killing of this study displayed different bactericidal characteristics from standard strain ATCC2592. This is because of its complicated origin background, so treatment of wild pathogens may call for a brand new dose regimen, like more frequent dosing intervals or large doses.

In the present investigation, three consecutive IMM CEQ administrations (75 mg/gland) produced an AUC of 42.25 mg·h/mL, the milk concentration vs. time curve is shown in Figure 3. The absorption of CEQ was rapid, drug reached its peak concentration at 1.8 h (*T*_max_ =1.8 h, *C*_max_ =1.55 mg/mL). This value was very similar to that in the cattle of Li's report (44.74 mg·h/mL, *T*_max_ =2 h, *C*_max_ =1.05 mg/mL) (34). The half-life of CEQ in this study was 10.6 h, which is similar to those described in cattle (6.3 h) following three consecutive IMM administrations (34) and in healthy lactating goats (12.9 h) (6).

According to a previous study, β-lactam antibiotics were time-dependent drugs (35) and showed bactericidal effect when the concentration in the target organ was above the MIC of the pathogen (36). According to previous research (30), %T>MIC and AUC_0−24_/MIC were the PK/PD index for CEQ (37). In this study, %T>MIC could not be obtained accurately. The AUC_0−24_/MIC in a previous study showed a good linear correlation with the antimicrobial effect of CEQ as the optimal PK/PD index (38). Thus, the PK/PD surrogate AUC_0−24h_/MIC was chosen as the optimal PK/PD index. The linear correlation coefficient of AUC_0−24h_/MIC and CEQ antimicrobial effect was higher for the initial inoculum of 10^6^ than 10^8^ CFU/mL (0.91 vs. 0.84). This result exhibited the impact of initial inoculum on PK/PD modeling result. The specific cause of impact needs further study. The EC_50_ of the 2014RQG-33.15 strain was 2,263 for the initial inoculum of 10^6^ CFU/mL, and the value of AUC_0−24h_/MIC for the bactericidal effect of ATCC29213 for the initial inoculum of 10^8^ CFU/mL was 12,266. This result was similar to that derived in an *in vivo* PK/PD integration in a lactating mouse model where the EC_50_ and AUC_0−24h_/MIC were 2,483 and 13,492, respectively (11), but the PK/PD parameters vary for other strains in this study. The possible reasons were as follows: (1) the PK/PD model is different. In this study, the *ex vivo* PK/PD model was used: previous work adopted the *in vivo* PK/PD model, and there was a distinction between the *ex vivo* and *in vivo* PK/PD models. (2) The dose of CEQ in mouse mammary gland tissues was lower than in the bovine gland. The concentration of CEQ mammary gland tissue was obtained that could be lower than in milk. Thus, the target animal and dose regimen was important for PK/PD modeling. (3) In this study, lactating bovine was used as a target animal rather than laboratory animals to reflect CEQ antimicrobial activity in clinical treatment.

Population dose prediction derived from MCS indicated that the existing dose of CEQ (55 mg/gland/12 h) was sufficient for cow mastitis caused by *S. aureus*. Under the recommended dose (55 mg/gland/12 h), even severely infected mastitis (10^8^ CFU/mL) could be cured, much less the slight infection mastitis (10^6^ CFU/mL). It was higher than that recommended in Shymaa's study (37.5 mg) (39) because in this study the severe infection (8 log_10_CFU/mL) was concerned. In addition, the recommended dose (55 mg/gland/12 h) was lower than the present using dose regimen (75 mg/gland/12 h) without reducing the antibacterial effect. Its application could reduce economic costs.

CO_PD_ is an important tool to set susceptibility breakpoint, and it was also used by regulatory agencies, such as EUCAST and VAST, to refine the susceptibility breakpoint (40–42). Monte Carlo simulation provides a great advantage using drug exposure–effect relationship (43), which considers pharmacokinetic variation in target animals, MIC distribution, and PK/PD indices in defining the PK/PD cutoffs. Currently, no breakpoint data of CEQ was established for animal infections caused by *S. aureus*. In the present study, the CO_PD_ of CEQ against *S. aureus* in bovine were determined to be 2 μg/mL at the recommended dose (55 mg/12 h/gland) and clinical dose regimen (75 mg/12 h/gland) based on Monte Carlo analysis, which was equal to the CLSI clinical CO_PD_ values of cefalotin (2 μg/mL), cefazolin (2 μg/mL), cefpodoxime (2 μg/mL), ceftiofur (2 μg/mL), and lower than cefoxitin (4 μg/mL) against *S. aureus* (15). However, the relatively conservative CO_PD_ should be verified in a larger number of bacteria and clinical practices.

Based on the current PK study, MIC distribution, and specific PD targets, if the dose is given at 55 mg/gland/12 h, the 10,000-subject Monte Carlo simulation showed that the bactericidal effect could be achieved under recommended dosage against *S. aureus* isolates in this study. CEQ 55 mg/gland/12 h is estimated to be effective against *S. aureus* infection in bovine. For the dose of 55 mg/gland/12 h recommended in this study, the PK/PD cutoff was 2 μg/mL. To confirm this value, CO_PD_ derived from *in vivo* PK/PD modeling should be investigated further in the future (40).

## Conclusion

This is the first study to assess *ex* vivo PK/PD of CEQ against *S. aureus*. (1) Milk as a medium can reflex the real mastitis progress in clinical therapy. (2) Initial bacterial concentration has a huge impact on antibacterial effect on all the tested three strains. (3) The *ex vivo* PK/PD based population dose prediction indicated a target attainment rate (TAR) of 90% of 55 mg/gland/12 h. (4) The PK/PD Cutoff value (CO_PD_) for CEQ against *S. aureus* was 2 μg/mL under the recommended dose of 55 mg/gland/12 h.

## Data Availability Statement

The raw data supporting the conclusions of this article will be made available by the authors, without undue reservation.

## Ethics Statement

The animal study was reviewed and approved by Jiangsu Administrative Committee for Laboratory Animals (SYXKSU-2007-0005).

## Author Contributions

XX and Z-qW conceived and designed the experiments, manuscript reviewing, and editing. L-jJ, K-xY, and TD performed the experiments. All authors contributed to the article and approved the submitted version.

## Funding

This project was supported by the National Natural Science Foundation of China (Nos. 31702291 and 31872526) and the Priority Academic Program Development of Jiangsu Higher Education Institutions, China.

## Conflict of Interest

The authors declare that the research was conducted in the absence of any commercial or financial relationships that could be construed as a potential conflict of interest.

## Publisher's Note

All claims expressed in this article are solely those of the authors and do not necessarily represent those of their affiliated organizations, or those of the publisher, the editors and the reviewers. Any product that may be evaluated in this article, or claim that may be made by its manufacturer, is not guaranteed or endorsed by the publisher.
