# Supplementary material for: Ex Vivo Pharmacokinetics and Pharmacodynamics Modeling and Optimal Regimens Evaluation of Cefquinome Against Bovine Mastitis Caused by Staphylococcus aureus
Source: Front Vet Sci. 2022 Mar 8;9:837882. doi: 10.3389/fvets.2022.837882 (PMC8957881; doi:10.3389/fvets.2022.837882)
Supplement: Supplementary file 1 [file Data_Sheet_1.docx]

**Table.S1 Primer sets for PCR 7 virulence genes**

| Gene | Sequence (5′ to 3′) | Amplicon size (bp) |
| --- | --- | --- |
| *luk* | F ATCATTAGGTAAAATGTCTGGACATGATCCA  R GCATCAASTGTATTGGATAGCAAAAGC | 433 |
| *nuc* | F GCGATTGATGGTGATACGGTT  R AGCCAAGCCTTGACGAACTAAAGC | 270 |
| *clf*A | F GGCAACGAATCAAGCTAATACAC  R TTGTACTACCTATGCCAGTTGTC | 719 |
| *fnb*A | F GCGGAGATCAAAGACAA  R CCATCTATAGCTGTGTGG | 1279 |
| *fnb*B | F GGAGAAGGAATTAAGGCG  R GCCGTCGCCTTGAGCGT | 812 |
| *hla* | F GGTTTAGCCTGGCCTTC  R CATCACGAACTCGTTCG | 534 |
| *hlb* | F GCCAAAGCCGAATCTAAG  R CGCATATACATCCCATGGC | 833 |
| *mec*A | F GTGAAGATATACCAAGTCATT | 147 |
|  | R ATGCGCTATAGATTGAAAGGAT |  |

**Table. S2 Distribution of the Minimal Inhibitory Concentration (MIC) of antimicrobials against *Staphylococcus aureus* (n = 63) isolated from Jiangsu China dairy farms**

| Antimicrobial  agent | ≤0.125 | 0.25 | 0.5 | 1 | 2 | 4 | 8 | 16 | 32 | 64 | ＞64 |
| --- | --- | --- | --- | --- | --- | --- | --- | --- | --- | --- | --- |
| STR | - | 1 | 1 | 6 | 9 | 15 | 7 | 11 | 7 | 3 | 2 |
| DOX | 3 | 34 | 8 | 11 | 2 | 1 | 1 | 3 | - | - | - |
| MEM | 33 | 23 | 6 | 1 | - | - | - | - | - | - | - |
| CIP | 19 | 36 | 7 | 1 | - | - | - | - | - | - | - |
| FFC | - | 0.25 | 5 | 10 | 19 | 26 | 2 | - | - | - | - |
| FOX | - | 3 | 1 | 2 | 2 | 20 | 12 | 9 | 7 | 7 | - |
| TET | 1 | 5 | 27 | 5 | 3 | - | 1 | 3 | 8 | 5 | 5 |
| APR | 1 | 1 | 2 | 7 | 10 | 13 | 13 | 13 | 3 | - | - |
| CLI | 6 | 9 | 3 | 25 | 9 | 3 | 1 | - | - | 7 | - |
| CEQ | 1 | 16 | 22 | 16 | 6 | 2 | - | - | - | - | - |
| CLO | 17 | 5 | 30 | - | 3 | 1 | 3 | 3 | 1 | - | - |
| PEN | 40 | 6 | 5 | 5 | - | - | 1 | 1 |  | 2 | 3 |
| CEQ in milk | 1 | 7 | 20 | 23 | 9 | 2 | - | 1 | - | - | - |

**Table.S3 In vitro susceptibility assays of 3 Staphylococcus aureus isolates used in *ex vivo* PK/PD modeling.**

| Strains | STR | DOX | MEN | CIP | FFC | FOX | TET | APR | CLI | CEQ | CLO | PEN | CEQin milk |
| --- | --- | --- | --- | --- | --- | --- | --- | --- | --- | --- | --- | --- | --- |
| B4048RH-31.4 | 16 | 1 | 0.5 | 0.125 | 2 | 64 | 16 | 4 | >64 | 4 | 0.5 | 1 | 16 |
| 2014RQG-33.15 | 1 | 0.25 | 0.125 | 0.06 | 1 | 0.25 | 0.5 | 0.25 | 1 | 1 | 0.5 | 0.06 | 2 |
| 29213 | 1 | 0.25 | 0.125 | 0.25 | 1 | 4 | 2 | 4 | 1 | 0.5 | 0.5 | 0.25 | 1 |

**Table.S4 Prevalence of virulence factor genes in *Staphylococcus aureus* strains isolated from bovine mastitis**

| Gene | No. of isolates | Prevalence (%) |
| --- | --- | --- |
| *pvl* | 4 | 6 |
| *clf*A | 23 | 37 |
| *fnb*A | 26 | 41 |
| *fnb*B | 28 | 44 |
| *hla* | 36 | 57 |
| *hlb* | 28 | 44 |

**Table.S5 MIC range and prevalence of MRSA and MIC50, MIC90 of 63 strains**

| MIC range of CFQ | MIC range in milk of CFQ | prevalence of MRSA | MIC_50_  (μg/mL) | MIC_90_  (μg/mL) |
| --- | --- | --- | --- | --- |
| 0.06-4 | 0.125-16 | 35/63 | 0.5 | 2 |


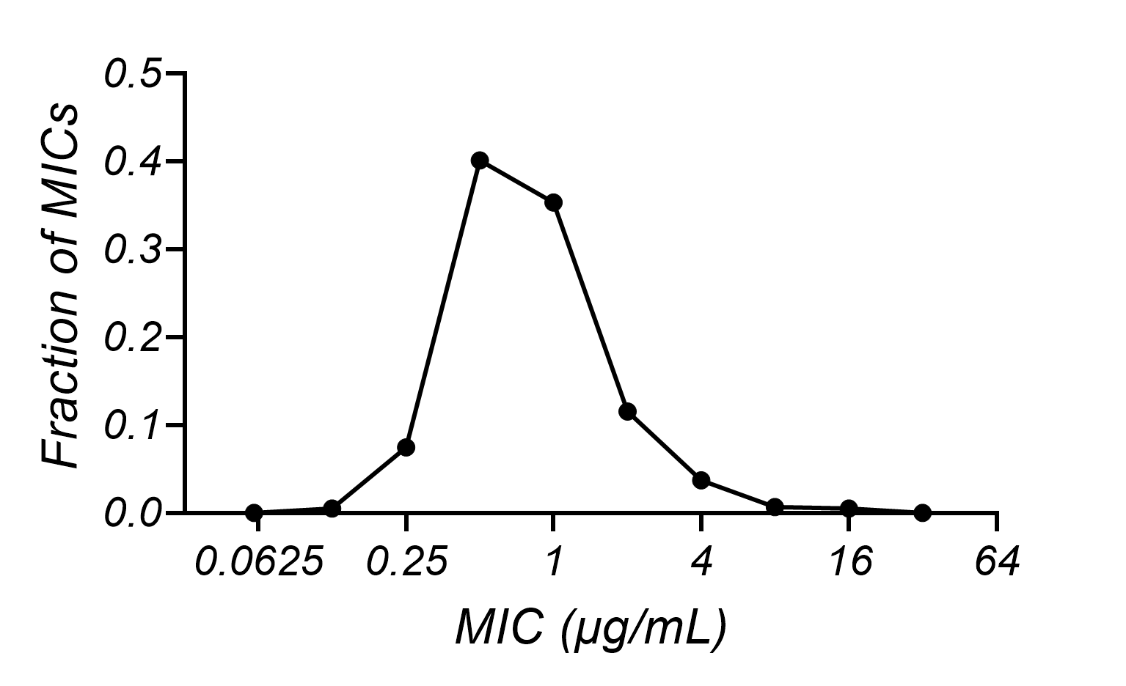


**Figure.S1 MIC distribution of cefquinome against *S. aureus* (n=588) in global**
